# Intravesical Recurrence of Upper Tract Urothelial Carcinoma: Strategies for Prevention

**DOI:** 10.1007/s11934-026-01331-6

**Published:** 2026-08-12

**Authors:** Lingmin Song, Margaret Meagher, Ava Saidian, Amirali Salmasi, Sarah Azari, Mai Dabbas, Aditya Bagrodia

**Affiliations:** 1 Department of Urology, Ningbo Hospital of Integrated Tranditional Chinese and Western Medicine, Zhejiang, China; 2 Department of Urology, University of California San Diego, San Diego, CA USA; 3 Department of Urology, University of Tennessee Health Sciences System, Memphis, TN USA

**Keywords:** Urothelial carcinoma, Bladder recurrence, Seeding

## Abstract

**Purpose of Review:**

This review focuses on intravesical recurrence (IVR) of upper tract urothelial carcinoma (UTUC).

**Recent Findings:**

We explore epidemiology, risk factors, mechanisms of tumor cell seeding, molecular pathways, genetic predispositions, and related factors of UTUC. Diagnostic techniques, strategies managing disease and reducing recurrence, as well as controversies and challenges in UTUC management are discussed.

**Summary:**

By synthesizing the latest research findings, this review aims to provide a comprehensive understanding of UTUC IVR which can guide clinical practice and future research efforts.

## Introduction

Intravesical recurrence (IVR) in the bladder is a significant clinical consideration for patients with UTUC. Indeed international guidelines advise periodic cystoscopy as a part of surveillance programs in patients with UTUC [1]. IVR may lead to multiple anesthetics in elderly and frail patients with associated delirium and adverse cognitive impacy. Further, there may be a need for radical cystectomy in approximately 10% of patients with IVR along with worse oncologic outcomes for advanced lower tract recurrences [2]. The reported incidence of IVR varies across different studies, mainly due to differences in patient populations, follow-up durations, and study designs. Bladder urothelial carcinoma (UC) presents synchronously or metachronously in up to 50% of patients with UTUC [3, 4]. A study of 482 patients with UTUC who underwent radical nephroureterectomy (RNU) found that the actuarial intravesical recurrence-free survival estimates at 2 and 5 years following surgery were 72% and 45%, respectively [3]. In a meta - analysis of 8275 patients, 29% were diagnosed with intravesical recurrence (IVR) within a median time to diagnosis of 22.2 months (range 6.7–56.5) [2]. Patients with a previous history of bladder cancer have been shown to be at a higher risk of IVR [3]. Identifying other predisposing characteristics can aid in developing more targeted surveillance and preventive strategies for patients at high risk of IVR.

## Pathophysiology and Risk Factors of IVR in UTUC

### Tumor Seeding

The seeding of tumor cells from the upper urinary tract to the bladder is one of the key mechanisms underlying IVR in UTUC. During surgical procedures such as diagnostic ureteroscopy or RNU, manipulated tumor cells may be dislodged and implanted in the bladder mucosa. In bladder cancer, transurethral bladder tumor resection (TUR-BT) is demonstrated to seed malignant cells into the bloodstream [5]. Similarly, in the context of UTUC, diagnostic ureteroscopy can potentially disrupt the tumor, leading to the release of tumor cells that may then seed the bladder.

Tumor-initiating cells (TICs) also provide another mechanism of the pathogenesis of UTUC. TICs possess self-renewal and other stem-cell properties, and are regarded as one of the causes of tumor initiation, recurrence, and metastasis [6]. In UTUC, TICs may be present in the tumor in the upper urinary tract. Tumor seeding in the bladder can occur when these cells are released either during surgical manipulation or in spontaneous tumor fragmentation. The presence of TICs in UTUC tumors may explain why some patients experience IVR even after what appears to be complete resection of the primary tumor in the upper urinary tract.

### Genetic Predispositions and Biomarkers

Genetic predispositions play a role in the development and recurrence of UTUC. Lynch syndrome, a hereditary DNA mismatch repair syndrome also implicated in uterine and colorectal cancer, is the most common genetic condition associated with UTUC [7]. Data suggests that individuals with UTUC in the context of Lynch syndrome are at an increased risk of IVR, highlighting the need to develop individualized protocols with escalated surveillance and perhaps adjuvant therapy [8].

IVR is a clonal disease, sharing identical molecular features to UTUC. Audenet and colleagues molecularly characterized 195 patients with UTUC using a targeted next generation sequencing (NGS) panel (MSK-IMPACT) [8]. 42% of the cases had metachronous lower tract recurrence. FGFR3 mutations, KDM6A mutations, and CCND1 mutations were associated with higher rates of bladder recurrence. In this study, Twenty nine of the patients had NGS performed on UTUC specimen and subsequent bladder recurrence—all bladder recurrences had a common clonal origin to the UTUC, suggesting a drop-metastasis mechanism of carcinogenesis.

Several biomarkers have been investigated for potential utility in predicting IVR. For example, endoglin expression in the urothelial cells of UTUC carcinomas has been associated with a higher risk of IVR. A study of 99 cases of primary UTUC treated with RNU found that endoglin expression was significantly higher in the endothelium of carcinomas than in paired benign urothelium, and was significantly associated with IVR when adjusting for other relevant clinicopathological variables (*p* = 0.015, HR = 2.67) [9]. Additionally, AIB1 and EIF5A2 expression have been shown to be independent predictors of IVR after RNU. In a study of 109 UTUC patients, high expression of AIB1 and EIF5A2 was associated with shortened bladder recurrence intervals, and in multivariate analysis, their expression along with pathologic nodal stage provided significant independent predictors for IVR(*p* = 0.034,HR = 3.252 and *p* = 0.022,HR = 4.613 respectively) [10]. If validated, these biomarkers can potentially be used to stratify patients and guide personalized treatment decisions.

### Tumor-related Factors

Tumor-related factors such as multifocality significantly influence the risk of IVR in UTUC. In a study of 177 patients with UTUC who underwent RNU, multifocality was identified as an independent predictor of IVR in both univariate and multivariate analyses(*p* = 0.026, HR = 4.40) [11]. Multifocal tumors may increase the likelihood of tumor cell dissemination during surgery leading to a higher risk of seeding in the bladder. Ureteric tumors have also been associated with a higher risk of IVR. A 30-year single-center experience study found that ureteric location was the only identified risk factor for bladder cancer recurrence after surgical management of UTUC (*p* = 0.002, HR not mentioned) [12]. Possible explanations for this include closer anatomic location facilitating tumor seeding either through the normal urinary flow or during surgical manipulation. Additionally, multifocal tumor in UTUC was also independent predictor of IVR (*p* = 0.01, HR = 1.75) and tumor multifocality may represent a higher potential for tumor cell shedding and seeding in the bladder. the presence of concomitant carcinoma in situ (CIS) in the upper urinary tract has been associated with IVR in a national multicenter study [3]. CIS may represent a more aggressive form of UTUC, with a higher potential for tumor cell shedding and seeding in the bladder.

### Impact of Diagnostic and Biopsy Approaches on IVR

Diagnostic approach for UTUC can have a significant impact on the risk of IVR. When UTUC is suspected based on axial imaging, cytology without biopsy, ureteroscopy, and percutaneous biopsy are available diagnostic tools. European Association of Urology guidelines for UTUC management promote ureteroscopy and cytology as initial diagnostic tools [1]. A positive cytology in the presence of imaging findings suggestive of UTUC and in the absence of lower tract disease, has a specificity of > 70% for diagnosis of UTUC. While imaging and cytology combined may be sufficient to begin treatment for UTUC, practitioners may prefer histopathologic confirmation, particularly when considering administration of neoadjuvant chemotherapy.

Diagnostic ureteroscopy (URS) prior to RNU is controversial. A systematic review and meta-analysis including 11 studies with 4,057 participants found that diagnostic URS prior to RNU significantly increased the IVR risk after RNU (HR 1.53, 95% CI 1.31–1.77; *p* < 0.001) [13]. This may be due to the mechanical disruption of the tumor during ureteroscopy, leading to the release of tumor cells that can seed the bladder. Douglawi and colleagues investigated the impact of ureteroscopy, stents, and access sheaths in 143 patients receiving RNU [14]. In this study, 104 patients received URS, 26 patients (25%) with access sheath and 45 patients (43%) with stent placement. The authors reported 11% IVR with access sheath, which approached the rate of IVR if no ureteroscopy was performed. The IVR with no sheath was 40%; further there was no difference in IVR rate with stent usage. These data suggest that access sheath should be considered when ureteroscopy is performed for renal pelvic tumors. The data is logical considering that shed tumor cells would directly exit the body via access sheath without interfacing with bladder mucosa.

Other diagnostic modalities such as percutaneous biopsy may not carry the same risk of bladder seeding that ureteroscopy does. Historic case reports of percutaneous biopsy of UTUC leading to tract seeding largely caused percutaneous biopsy to be avoided in suspected cases of UTUC. More recently, a single-institution series and updated meta-analysis found that percutaneous biopsy before RNU was safe and associated with lower rates of IVR in comparison to ureteroscopy [15]. It is important for clinicians to be aware of advantages and limitations of the various diagnostic techniques in patients with UTUC.

## Prevention Strategies for Intravesical Recurrence in UTUC

There are multiple point-of-care opportunities at the time of RNU that can lower risk of IVR. It is critical that the team considers and implements these practices for this relatively uncommon operation.

### Early Clipping of the Ureter

Early ligation of the ureter during radical nephroureterectomy has been investigated as a potential strategy to reduce IVR preventing the flow of tumor cells to the bladder before surgical manipulation of the tumor. Early clipping of the ureter prior to hilar dissection and kidney mobilization mechanically obstructs shed tumor cells from entering the bladder. A prospective single-arm multicenter clinical trial evaluated the utility of early ureteral ligation in preventing intravesical recurrence after RNU for upper urinary tract urothelial carcinoma [16]. The results showed that in patients with renal pelvis cancer, early ureteral ligation decreased the rate of intravesical recurrence, with 1 - and 2 - year intravesical recurrence - free survival rates of 89% and 86% in the early ligation group compared to 74% and 64% in the control group (*p* = 0.025) [16]. A systematic review and meta-analysis of 31 studies found that early ligation (EL) of the ureter was associated with a significantly lower rate of IVR compared to those who did not have early ligation (HR: 0.64, 95% CI: 0.44–0.94, *p* = 0.02) [17]. We suggest that a clip be placed on the ureter immediately following medialization of the bowel as a very simple means of reducing IVR.

### Management of the Bladder Cuff

Multiple techniques including pluck technique, transurethral resection, stripping, and bladder cuff removal are described to manage the intramural ureter. Complete bladder cuff removal is superior to the remainder of described techniques to minimize intravesical recurrences [18]. RNU with bladder cuff resection remains standard of care for certain patients with UTUC [19]. The optimal surgical approach to reduce intravesical recurrence has been a subject of considerable research and debate. Open and minimally invasive approaches to RNU are both commonly used. Minimally invasive RNU is attractive due to improved convalescence in patients undergoing multiquadrant surgery [17]. Excision of the bladder cuff with distal ureter can be technically challenging using pure laparoscopic approaches and can also be difficult with the assistance of robotic platforms. A systematic review and meta-analysis of 31 studies on RNU found that laparoscopy significantly increased the intravesical recurrence compared to open techniques (HR: 1.28, 95% CI: 1.06–1.54, *p* < 0.001) [17]. Comparing surgical modalities, laparoscopic RNU was associated with an increased risk of IVR compared to open RNU [17]. The reasons for this may include differences in the surgical field, handling of the specimen, and potential for tumor cell dissemination during laparoscopic procedures. Minimizing urine and tumor spillage when minimally invasive techniques are used is mandatory.

If minimally invasive RNU is to be employed, we recommend completely emptying the bladder prior to cystotomy, early clipping of the ureter, mobilization of intravesical ureter so complete bladder cuff can be excised, and anterior cystotomy to visualize resection of actual ureteral orifice. Furthermore, the increased risk of IVR with minimally invasive approaches should be discussed with patients receiving RNU. As larger experience with minimally invasive RNU accrues along with maneuvers to decrease IVR, more contemporary data is warranted.

### Perioperative Instillation of Intravesical Chemotherapy

Level 1 data establishes a clear role for single-dose perioperative administration of intravesical chemotherapy at the time of RNU to minimize risk of lower tract recurrence. Two prospective clinical trials and subsequent metanalyses establish the role of instilling mitomycin-C after RNU [20–22]. In the ODMIT-C trial, instillation of mitomycin-C was associated with an 11% absolute risk reduction of recurrence [20]. Similarly, the REBACARE trial found that peri-operative instillation of mitomycin-C was also associated with a reduced risk of recurrence in patients who had not undergone previous diagnostic ureteroscopy [21]. Intravesical instillation of pirarubicin has also been associated with decreased risk of IVR [23]. There is considerable heterogeneity with respect to timing of intravesical chemotherapy in the above trials, with administration varying from 2 to 10 days. Despite the proven efficacy of these chemotherapeutic agents, contemporary data suggests utilization by urologic oncologists is only 51% [24]. Due to concerns of tissue necrosis associated with extravesical leakage of mitomycin-C, exorbitant cost of mitomycin-C, and questions regarding timing of instillation, gemcitabine has been investigated as an alternative agent. Retrospective evidence suggests that IVR following gemcitabine instillation is comparable to historic rates following mitomycin-C instillation [25], while prospective evidence is forthcoming. We endorse a single dose instillation of gemcitabine at the time of RNU whereby medication is instilled into the bladder for one hour at the start of the case and then subsequently drained from the bladder.

While similar high quality data does not exist for patients receiving diagnostic or therapeutic ureteroscopy, it is reasonable to believe that peri-procedural instillation of intravesical chemotherapy can reduce lower tract recurrences. Prospective trials investigating this clinical question are needed.

### Role of Systemic Chemotherapy and Immunotherapy in Recurrence Prevention

Systemic chemotherapy and immunotherapy also impact IVR in UTUC. A retrospective study of 133 patients with locally advanced disease defined by pathological T stage ≥ 3 UTUC carcinoma or nodal involvement found that adjuvant systemic chemotherapy was significantly associated with a lower risk of IVR [26]. The 1-year IVR-free survival rates of patients with and without adjuvant systemic chemotherapy were 86.0% and 70.2%, respectively (*p* = 0.046). While an interesting clinical observation, this is not clinically actionable since decision to administer adjuvant chemotherapy is dictated by mortality risk.

## Conclusion

IVR following nephrouterectomy represents a significant source of morbidity and mortality for patients with UTUC. At present, several surgeon-controlled factors in diagnosis and management can significantly impact the risk of IVR. There are several opportunities for decreasing IVR, including the use of optimal diagnostic and biopsy methods, appropriate surgical techniques, and adjuvant intravesical therapies. Future research should focus on decreasing IVR by developing more accurate predictive models using a combination of genetic, biomarker, and clinical factors, refining treatment and surveillance protocols, and exploring novel intravesical and systemic agents.

## Key References

- Fujita K, Ujike T, Nagahara A, Uemura M, Tanigawa G, Shimazu K, Fushimi H, Yamaguchi S, Nonomura N. Endoglin expression in upper urinary tract urothelial carcinoma is associated with intravesical recurrence after radical nephroureterectomy. Int J Urol. 2015 May;22(5):463-7. doi: 10.1111/iju.12719. Epub 2015 Jan 29. PMID: 25631522.
  - ○ Review of mechanism of seeding.
- Yamashita S, Ito A, Mitsuzuka K, Ioritani N, Ishidoya S, Ikeda Y, Numahata K, Orikasa K, Tochigi T, Soma F, Namima T, Arai Y. Efficacy of early ureteral ligation on prevention of intravesical recurrence after radical nephroureterectomy for upper urinary tract urothelial carcinoma: a prospective single-arm multicenter clinical trial. Jpn J Clin Oncol. 2017 Sep 1;47(9):870-875. doi: 10.1093/jjco/hyx085. PMID: 28903527.
  - ○ Elucidation of important prevention strategy.

## Data Availability

No datasets were generated or analysed during the current study.

## Declarations

### Human/Animals Study

This article does not contain any studies with human or animal subjects performed by any of the authors.
